# Sensory Perception: An Overlooked Target of Occupational Exposure to Metals

**DOI:** 10.1155/S1565363303000165

**Published:** 2003

**Authors:** Fabriziomaria Gobba

**Affiliations:** Cattedra di Medicina del Lavoro Dipartimento di Scienze Igienistiche Università di Modena e Reggio Emilia Via Campi 287 Modena (MO) 41100 Italy

## Abstract

The effect of exposure to industrial metals on sensory perception of workers has received only modest interest from the medical community to date. Nevertheless, some experimental and epidemiological data exist showing that industrial metals can affect vision, hearing and olfactory function, and a similar effect is also suggested for touch and taste. In this review the main industrial metals involved are discussed. An important limit in available knowledge is that, to date, the number of chemicals studied is relatively small. Another is that the large majority of the studies have evaluated the effect of a single chemical on a single sense. As an example, we know that mercury can impair hearing, smell, taste, touch and also vision, but we have scant idea if, in the same worker, a relation exists between impairments in different senses, or if impairments are independent. Moreover, workers are frequently exposed to different chemicals; a few available results suggest that a co-exposure may have no effect, or result in both an increase and a decrease of the effect, as observed for hearing loss, but this aspect certainly deserves much more study. As a conclusion, exposure to industrial metals can affect sensory perception, but knowledge of this effect is yet incomplete, and is largely inadequate especially for an estimation of “safe” thresholds of exposure. These data support the desirability of further good quality studies in this field.


### Sensory Perception: An Overlooked Target of Occupational Exposure to Metals.

Fabriziomaria Gobba

*Cattedra di Medicina del Lavoro,*
*Dipartimento di Scienze Igienistiche,*
*Università di Modena e Reggio Emilia, Modena (MO), Italy*

(Received January 26, 2003; Revised February 21, 2003; Accepted March 13, 2003)

**ABSTRACT**

The effect of exposure to industrial metals on sensory perception of workers has received only modest interest from the medical community to date. Nevertheless, some experimental and epidemiological data exist showing that industrial metals can affect vision, hearing and olfactory function, and a similar effect is also suggested for touch and taste. In this review the main industrial metals involved are discussed. An important limit in available knowledge is that, to date, the number of chemicals studied is relatively small. Another is that the large majority of the studies have evaluated the effect of a single chemical on a single sense. As an example, we know that mercury can impair hearing, smell, taste, touch and also vision, but we have scant idea if, in the same worker, a relation exists between impairments in different senses, or if impairments are independent. Moreover, workers are frequently exposed to different chemicals; a few available results suggest that a co-exposure may have no effect, or result in both an increase and a decrease of the effect, as observed for hearing loss, but this aspect certainly deserves much more study. As a conclusion, exposure to industrial metals can affect sensory perception, but knowledge of this effect is yet incomplete, and is largely inadequate especially for an estimation of "safe" thresholds of exposure. These data support the desirability of further good quality studies in this field.

**Key Words:** Toxic Metals, Occupational Exposure, Hearing, Smell, Taste , Touch, Vision.

---

Send communications to:
Fabriziomaria Gobba (MD)
Cattedra di Medicina del Lavoro, Dipartimento di Scienze Igienistiche
Università di Modena e Reggio Emilia, Via Campi 287; 41100 Modena (MO), Italy
Tel. +39 059 205 5463; Fax +39 059 205 5483; e-mail f.gobba@unimore.it

### INTRODUCTION

The possible impairment in sensory perception induced by occupational exposure to some metals is not unknown: the first description of human ocular effects of organic mercury was as early as 1885 /1/ and, in the same period, hearing loss was recognized among the signs of acute and chronic mercury poisoning in laboratory assistants preparing dimethylmercury amalgams /2/. Furthermore, in the first half of the 1900s anosmia was observed in cadmium exposed workers /3/. Nevertheless, despite these observations, the effect of toxic metals on sense organs and sensory perception in exposed workers has received relatively scant attention from the medical community to date, and available scientific literature is mainly limited to case-reports or isolated studies and, with a few exceptions, there has been no concerted effort in the field.

In this paper we provide an overview of current knowledge on the effect induced by industrial metal exposure to sensory perception in the hearing, smell, taste, touch and vision systems. In principle, these effects can be studied in animals or in humans, following experimental exposure, pharmacological treatments, occupational or environmental exposure: this review is mainly confined to the results of research on occupational exposure in workers but, when opportune, some other studies were also included.

### HEARING

Compared to the other senses, a relatively larger body of data exists on the effect of industrial toxic metals on hearing function of workers. This difference is likely to be due to a better consensus among researchers on testing procedures to be applied in research on exposed workers: in the large majority of studies pure tone audiometry and, less frequently, brainstem auditory evoked responses (BAER) were carried out, even if a few researchers also applied other procedures. Furthermore, a standardization of methods has been proposed, and the results reported by different research groups are, usually, reasonably comparable.

Overall results show that exposure to several industrial metals can affect hearing function. The main metals affecting hearing in occupational exposed workers are lead, manganese and mercury /4/. Lead can affect hearing function at low occupational exposure level /5, 6, 7, 8/, while manganese seems able to induce this effect only in workers presenting other symptoms of intoxication /9/. In the second half of the nineteenth century, hearing loss was described among the signs of acute and chronic mercury poisoning in laboratory assistants preparing dimethylmercury amalgams /2/, but recent studies on hearing function in groups of workers exposed to organic mercury are lacking. A dose-related elevation in hearing threshold /10/, and an effect on BAER /11/ have recently been reported in occupational exposure to inorganic Hg. Considering environmental (non occupational) exposure, an impairment in hearing was diagnosed in up to 80% of patients with Minamata disease /12/, and a significant arsenic-related hearing loss was observed in children living in a heavily polluted area /13, 14/. Environmental lead exposure was also associated with a minor hearing impairment /15, 16/, even though a few non-positive results have been published /17, 18, 19/. Furthermore, experimental data in animals show that cadmium, platinum and tin can also exert an ototoxic effect /4, 20/.

Knowledge on pathogenetic mechanisms of metal ototoxicity is certainly far from complete, nevertheless some possible mechanisms were proposed. In arsenic experimental exposure histopathologic damage in the Corti organ and in stria vascularis were observed /2/. In sodium arsenilate intoxication, outer hair cells of helicotrema are affected first, then damage proceeds basally, and the progression seems time- and dose-dependent; inner cells seem more resistant /21/. Interestingly, the experimental finding of an early apical involvement of Corti organ is consistent with audiometric results in exposed populations, showing low-frequency sensory hearing loss /13, 14/. A similar involvement of the Corti organ was also suggested for an industrial solvent, toluene: in exposed rats, histopathological studies show a broad loss of outer cells in both mid- and mid-apical coil, consistent with electrophysiologic data showing a hearing deficit in mid- and mid-low frequency region /22/. This effect may be the result of an elevation in free intracellular calcium in hair cells directly produced by toluene /23/. Limited information is available on mechanisms in toluene exposed workers, but results reported by Morata *et al.* /24/ suggest retrocochlear damage. The case of lead is different: evidence from both animal and human studies suggest segmental demyelination and axonal degeneration of the eighth nerve induced by this metal /25/, while cochlear structure, including sensory cells, appears not affected /4, 25/. An effect of low level lead exposure on the central auditory nervous system was reported in exposed workers /5/. Still different is the example of mercury: data from Minamata disease affected patients suggest that early and middle stages of intoxication may induce cochlear lesions, whereas in late stages the retrocochlear part of auditory system is involved /26/. Demyelination in the temporal lobes and heavy deposition of the metal in transverse temporal gyri was observed in brain autopsy from mercury intoxicated subjects /26/. Atrophy of the cerebral and cerebellar cortices was also reported /27/. The hearing loss seems to develop quite early in intoxication, and extends over almost the entire frequency range /4/; similar findings were also observed in intoxicated monkeys /28/.

Scanty data are available on the reversibility of metal-related hearing loss, but a progressive increase of the effect was reported in experimental Hg exposure in monkeys /28/, and is suggested by some results obtained in metal exposed workers /4/, and in environmental Hg exposure /29/. Children seem more susceptible to the ototoxic effect of lead according to the results of some studies /16/, but not to others /17, 18/.

### SMELL

Smell is the perception of odour by the nose /30/. Receptors are located in a relatively unprotected position; as a consequence, it is not surprising that olfactory perception may be affected by airborne pollutants /31/. Damage to the olfactory neuroepithelium and bulb, or a functional impairment were observed in various experimental studies in animals /32, 33/ and some scattered data also exist in exposed workers. A review of the studies on the effect of industrial chemicals on smell is problematic. The first difficulty is the lack of an agreement among researchers on testing procedures /30, 34, 35, 36/. Odour detection threshold, discrimination or identification were most frequently adopted for studies in groups of workers /37, 38, 39, 40, 41, 42, 43, 44/, but the prevalence of subjective smell disturbance was evaluated in others /45/. Commercially available methods such as the "University of Pennsylvania Identification Test" (UPSIT) /46/ or the "Connecticut Chemosensory Clinical Research Center Test" (CCCRCT) /47/ were frequently applied in studies performed in the USA, while in Europe, the "Sniffin' Sticks" test battery, based on the Kobal and Hummel method /36/, or Elsberg and Levy's blast injection method modified by Pruszewicz /48/, or a combination of different methods /41, 49/ were most commonly adopted. In addition, odours included in batteries prepared in the USA and in Europe are different, and some adaptation is necessary for application in Europe of USA batteries, and vice versa. To avoid this problem, a Cross-Cultural Smell Identification Test has been developed and standardized in a number of countries /50/. The reliability of different olfactory tests for an evaluation of olfactory function was studied in volunteers, and the results suggest some caution in the comparison of results obtained using different methods /51/. Demographic and personal characteristics such as gender, age and cigarette smoking are significantly related to olfactory function /35, 37, 46,/.
Several chemicals, including pharmaceutical drugs, were reported to impair olfactory function in humans (for a review see /52/ and, more recently, /53/), but only industrial chemicals that can induce hyposmia or, less frequently anosmia, or dysosmia in exposed workers are considered here. The best known is certainly cadmium: a description of anosmia in exposed workers was first reported at the end of the forties /3/. In the following years an impairment in olfactory function related to cadmium exposure was observed in alkaline batteries workers, smelters, solders, and brazing workers /3, 40, 42, 44, 54, 55, 56, 57, 58, 59, 60/. Nevertheless also other metals, like chromium and nickel, have been reported inducing hyposmia or anosmia in workers /52, 58, 59/ Similar effect may be induced by several organic compounds /38, 39, 45, 52, 58, 59, 61, 62, 63, 64, 65/.

Knowledge on mechanisms of chemical related olfactory impairment is far from complete. Possible sites of action include the olfactory mucosa, the olfactory receptor site, the primary olfactory neuron, the olfactory bulb and the olfactory cortices (pyriform, prepyriform and entorhinal). Probably, different pathogenetic mechanisms are involved depending on the chemical: e.g. an effect on neurotransmitter release, or an interference with calcium channels in the nerve ending was postulated for cadmium /40, 66/, but other mechanisms are also possible /40, 42/. An olfactory uptake of metals was also described /67/.

Occupational-related olfactory impairment may be frequently subclinical /53/. Data on the course of this effect are scant.

### TASTE

Taste can be defined as the perception of salty, sweet, sour or bitter by specialized receptors, namely the taste buds. The majority of taste buds are in the tongue, but they are also in the soft palate, pharynx, larynx, epiglottis, uvula and first third of the esophagus /30, 35, 68, 69/. Saliva also plays an important role in gustatory function. Gustatory information is carried to the medulla oblongata in the brainstem by three cranial nerves: VII, IX and X, but fibers of the V nerve can also be stimulated by high concentrations of some gustatory stimuli /34/. Due to the superficial placement, taste buds are susceptible to direct chemical injury. Taste is also affected by changes in the composition and quantity of saliva /70/. Disorders of taste include a diminished sensitivity (*hypogeusia*), an altered perception (*dysgeusia*), a taste sensation in apparent absence of gustatory stimulus (*phantogeusia*) or a complete absence of gustatory perception (*ageusia*). In the gustatory system, fundamentally different transduction sequences underlie the perception of the different taste qualities and, at least for bitter, that of different compounds with the same quality; accordingly hypogeusia can be the result of both a total, but isolated, loss in sensitivity to compounds eliciting a specific quality, or a more generalized loss of sensitivity to compounds inducing a variety of taste qualities. Furthermore, even a completely ageusic subject may detect high concentrations of some gustatory stimuli through stimulation of trigeminal nerve fibers /34, 35/.

A consequence of the complexity is that clinical assessment of taste is less developed and standardized compared to other senses like smell or hearing /34/; for a review of many of the procedures used in clinical practice see Gent *et al.* /71/. Taste sensitivity declines with age /72/.

For all the cited reasons, it is not surprising that less knowledge is available on chemical induced taste disorders, and on its mechanisms, compared to other senses. Several pharmaceutical drugs have been implicated in altered taste sensations, representing one of the most common etiological factors of acquired dysfunction /35, 69, 70/, but very few studies have addressed the effect of industrial chemicals in exposed workers. An increase in taste threshold has been reported in chromium workers /73/. Pantogeusia has been associated with industrial or waste-site exposure to toxic levels of metals as cadmium, lead and mercury /74, 75/. Furthermore pantogeusia is a well known symptom of metal fume fever /76, 77/. In the most part of studies on taste disorders in workers, the presence of subjective disturbances were considered, while a few authors tested thresholds in taste qualities /73/.

Some more information on the possible effect of metals on taste can be obtained from experimental exposure in animals: mercury and cadmium can induce structural alteration in taste buds of fish /78, 79/. Furthermore, Cu, Hg and Zn can depress response to some taste stimulants /32, 80/.

Possible mechanisms involved in the pathogenesis of taste disorders related to exposure to industrial chemicals are largely unknown. Since heavy metals can be concentrated in saliva, and since taste responses can be altered or blocked by topical application of heavy metal solutions, this mechanism was proposed to explain the effect observed in exposed workers /81/. Nevertheless, other mechanisms, such as an alteration of salivary constituents, disruption of transduction/receptor mechanisms or also an alteration in the central processing of gustatory input, are also possible /34/.

The course of the effect in metal exposed workers is unknown, while, at least in the case of taste disorders related to occupational solvent exposure, some reversibility seems possible /45/.

### TOUCH

The definition of touch is somewhat more difficult compared to other senses. When we see or hear a particular event, it is obvious which sensation, sense organs, and receptor populations are involved, while with touch it is not so: tactile sensation includes both "passive" and "active" components /82/, includes components such as the perception of pressure, changes in temperature, pain and other, from different receptors, and involving different nerve fibres /83/. Furthermore, while the hand is the quintessential tactile organ, tactile sensations may also be elicited from the remainder of the body surface. A discussion on the complex components of touch, and of its anatomo-physiological aspects, is beyond the objectives of this review.

Vibration perception threshold (VPT), two-point discrimination, depth sense perception, temperature threshold, pain threshold and other procedures can be applied for research in groups of workers; nevertheless VPT is, in general, the most commonly adopted. Usually hand or toe thresholds or, more frequently, both are tested. Several testing devices and protocols for quantitative evaluation of perception threshold have been proposed /84, 85, 86, 87, 88, 89, 90, 91, 92/, and reference values reported /87, 88, 91, 92, 93, 94/. Various demographic and personal characteristics such as gender, age, height, skin temperature, alcohol consumption, and diseases such as diabetes, carpal tunnel syndrome and others can interfere with VPT /87, 88, 91, 92, 93, 94,95/. Some variability of results among authors exists, possibly related to differences in equipment and testing procedures.

Knowledge on pathogenetic mechanisms of VPT impairment induced by industrial chemical exposure is insufficient. An impairment in VPT is considered a sensitive indicator of an effect to the peripheral nerve, but an involvement in peripheral receptor mechanisms was suggested by some neurophysiological studies /96, 97, 98/. The effect may also be related to both receptor and neuron involvement. Vibration sensations are conveyed by large myelinated fibres; accordingly VPT gives scant information on smaller fibres, frequently involved in peripheral neuropathies /91/.

In occupational medicine, VPT measurement has been largely applied in research on the effect of hand-arm vibrations, and of repetitive strain /99, 100/, whereas a few studies have evaluated the effect of exposure to industrial chemicals. An effect on VPT was observed in lead exposure /89/, at relatively low levels too /98/. VPT measures have been applied for diagnosis of the subclinical effects of environmental exposure to elemental mercury /101/; environmental exposure to mercury was reported to affect also two point discrimination /102/. An effect on VPT was also reported in workers exposed to various solvents /97, 103, 104, 105, 106, 107, 108/ and to some pesticides /96, 109, 110, 111/.

Chemical related VPT impairment can be subclinical, as evidenced in lead exposure /98/. No data are available on the progression of this effect.

An association of VPT testing with other procedures, such as pin-prick sensitivity or temperature threshold, in studies in exposed workers would add relevant information on the effect of chemicals on tactile perception, but very few efforts have been made in this ambit.

### VISION

Like touch, vision is a complex perception, involving various different components. The anatomo-physiological aspects are not faced here, since this review is limited to studies on the effect of occupational exposure to industrial metals.

The main methods that can be applied for testing visual function in groups of workers are visual evoked potentials (VEPs), visual contrast sensitivity (VCS), color vision testing and also visual field /112, 113, 114, 115, 116/. Several other methods, such as the electroretinogram, are also available, but for practical reasons their use in an occupational setting has been very limited.

Various demographic and personal characteristics like age, alcohol consumption, smoking, and diseases like diabetes, hypertension, low vision and other eye diseases, and drugs interfering with the nervous system can interfere with the results of testing /114, 117/.

Several studies were performed on the effect of various industrial chemicals on visual function in exposed workers /114, 115/, but relatively few data are available on metals. In workers exposed to levels close to the current occupational limits, mercury can induce an impairment in color vision /118, 119, 120, 121, 122, 123/; an environmental exposure to the same metal may impair VCS /124, 125, 126/. Lead may also affect VCS in workers /128/, whereas VEPs results are conflicting /129, 130/; again, children seem more susceptible /131/. An effect on VEPs was reported in workers exposed to manganese /132/, while data on occupational exposure to other metals are lacking. Color vision, VEPs, and VCS are also affected by occupational exposure to various solvents and to some pesticides (for a review see /114/, and /115/).

The chemical-related visual effects in workers are usually subclinical, and color vision testing, VEPs and VCS can disclose early effects of chemical exposure. The course is discussed: regarding color vision a progression related to an increase in exposure was observed in solvent exposed workers, while data on reversibility are conflicting /133, 134, 135/. Insufficient data are available on VEPs testing, but a recent study suggests that, at least in styrene exposure, contrast sensitivity loss may reflect long-term cumulative exposure and chronic damage, possibly irreversible, to the neuro-optic pathways /136/.

Knowledge on mechanisms of chemical related impairment in visual perception is largely insufficient. Available data come mainly from solvents exposure: retinal location is suggested by some electrophysiological data in workers exposed to styrene and perchloroethylene /137/ and in experimental exposure to various solvents /138/. The effect may be related to a direct effect of the chemical, or of metabolites, on receptor functioning (e.g. on membrane of cone), or on neurotransmitters like dopamine /139/. Another possibility is an axonopathy of the optical pathway /140/ but other pathogenetic mechanisms cannot be ruled out.

### CONCLUSIONS

As a whole, results from studies reported in this review show that several industrial metals can impair sensory functions in the hearing, smell, taste, touch and vision systems; a list of the relevant metals is presented in Table 1. Furthermore, some studies were published showing the possibility that also maternal exposure to industrial chemicals during pregnancy can result in an impairment of sensory perception in offspring /131, 141, 142, 143/. Nevertheless to date knowledge in this field is still largely incomplete and fragmentary. One of the main causes is certainly the lack of agreement on testing methods; this is supported by the observation that, where standardized methods exist, as is the case of pure tone audiometry for hearing

**Table 1**

Studies reporting effect of industrial metals on sensory perception.

| | HEARING | SMELL | TASTE | TOUCH | VISION |
|---|---|---|---|---|---|
| **Arsenic** | Bencko *et al*, 1977a; 1977b (1)<br>Rybak, 1992 (3); | | | | |
| **Cadmium** | Rybak 1992, (2, 3)<br>Whitworth *et al*, 1999 (2); | Friberg, 1948; 1950;<br>Adams, 1961;<br>Potts, 1965;<br>Liu *et al*, 1985;<br>Rose *et al*, 1992;<br>Duncan and Smith, 1995<br>Rydzewski *et al*, 1998;<br>Klimek *et al*, 1999 (3);<br>Suruda, 2000;<br>Sulkowski *et al*, 2000<br>Mascagni *et al*, 2002; | Henkin, 1975 (3);<br>Borovyagin *et al*, 1989 (2); | | |
| **Chromium** | | Amoore, 1986 (3);<br>Duncan and Smith, 1995;<br>Klimek *et al*, 1999 (3). | Seeber and Fikentscher, 1990 | | |

Data are mainly confined to the effect of occupational exposure in workers, nevertheless if necessary some studies on environmental exposure, or on experimental exposure in animals, have also been included.

(1): environmental exposure; (2) experimental results in animals; (3) review study.

**Table 1** (continued)

Studies reporting effect of industrial metals on sensory perception.

| | HEARING | SMELL | TASTE | TOUCH | VISION |
|---|---|---|---|---|---|
| **Copper** | | Baatrup, 1991 (2) | Iwasaki and Sato, 1984 (2, 3);<br>Baatrup, 1991 (2; 3); | | |
| **Lead** | Schwartz and Otto, 1991 (1);<br>Araki *et al*, 1992;<br>Rybak, 1992 (3);<br>Otto and Fox., 1993 (1);<br>Farahat *et al*, 1997<br>Forst *et al*, 1997;<br>Osman *et al*, 1999 (1);<br>Wu *et al*, 2000; | | Henkin, 1975 (3); | Kovala *et al*, 1997<br>Chuang *et al*, 2000 | Otto and Fox., 1993<br>Abbate *et al*, 1995<br>Murata *et al*, 1995<br>Lucchini *et al*, 2000<br>Rothenberg *et al*, 2002 |
| **Manganese** | Nikolov, 1974; | Amoore, 1986 (3);<br>Duncan and Smith, 1995; (2) | | | Sinczuk-Walczak *et al*, 2001 |
| **Mercury** | Kurkland *et al*, 1960 (1);<br>Miller, 1985 (3);<br>Rybak, 1992 (3);<br>Pawlas, 2002; | Amoore, 1986 (2,3).<br>Baatrup, 1991; | Henkin, 1975 (3);<br>Borovyagin *et al*, 1989 (2); | Mergler, 1995<br>Harada *et al*, 2001 | Langauer-Lewowicka and Kazibutowska, 1989<br>Ellingsen *et al*, 1993<br>Cavalleri *et al*, 1995; 1998<br>Gobba *et al*, 1996<br>Hudnell *et al*, 1996 (1)<br>Lebel *et al*, 1996; 1998 (1).<br>Altmann *et al*, 1998;<br>Urban *et al*, 1999;<br>Urban *et al*, 2002 |

(1): environmental exposure; (2) experimental results in animals; (3) review study.

**Table 1** (continued)

Studies reporting effect of industrial metals on sensory perception.

| | HEARING | SMELL | TASTE | TOUCH | VISION |
|---|---|---|---|---|---|
| **Nickel** | | Amoore, 1986 (3);<br>Duncan and Smith, 1995 (3);<br>Klimek *et al*, 1999 (3); | | | |
| **Platinum** | Rybak 1992, (2, 3)<br>Blakeley and Myers, 1993; | | | | |
| **Tin** | Rybak 1992, (2, 3)<br>Fechter and Carlisle, 1990 (2); | | | | |
| **Zinc** | | | Iwasaki and Sato, 1984 (2,3);<br>Baatrup, 1991 (2; 3); | | |

(1): environmental exposure; (2) experimental results in animals; (3) review study.

testing, or that of color vision testing /114/, a better knowledge exists. An important limitation in available data is that, in the large majority of the studies published to date, the effect of a single chemical on a single sense was evaluated: as an example, we know that lead exposure can impair hearing, taste, touch and vision but we have scant idea if, in the same worker, a relation exists between impairments in different senses, or if impairments are independent. Moreover, a simultaneous exposure of workers to different chemicals is very frequent: a few results suggest that a co-exposure may have no effect, or result in both an increase and a decrease of the effect, as was observed for hearing loss /20, 144, 145/, but data are largely incomplete.

Last, but not least, knowledge on pathogenetic mechanisms of metals-related sensory impairments is largely insufficient, and the possible thresholds of exposure for induction of the effect on sensory perception are unknown.

Accordingly, further good quality research on the effect of occupational exposure to chemical on sensory perception is certainly needed, and justified.

This is a complex task, and is first of all based on the possibility of an improvement of the method, including an agreement and a standardization of procedures for quantitative testing of perception, better information on interfering factors, and an evaluation of reference values for non exposed groups.

The improvement of the method is the necessary base for further studies on the effect of chemicals not or scantily studied, of co-exposure to different chemicals, and on the overall comprehensive effect on all the senses.

The subsequent steps of this research are:

1. Studies on the possible pathogenetic mechanisms;
2. Evaluation of possible thresholds.

This course is certainly difficult, and deserves a coordinated effort of different research groups and adequate resources; nevertheless it seems justified by the ultimate result: the possibility to prevent an avoidable part of sensory perception impairment, an important cause of the decline in quality of life, especially in old age.

### REFERENCES

1. G.N. Edward, *St Bart's. Hosp. Rep.* **1**, 141 (1885).
2. J.J. Miller, In: *Handbook of Ototoxicity,*. Boca Raton, Fla, CRC Press, 1985; 237.
3. L. Friberg, *J. Ind. Hyg. Toxicol.* **30**, 32 (1948).
4. L.P. Rybak, *Otolaryngol. Head Neck Surg.* **106**, 677 (1992).
5. S. Araki, K. Murata, K. Yokoyama and E. Uchida, *Am. J. Ind. Med.* **21**, 539 (1992).
6. T.M. Farahat, G.M. Abdel-Rasoul, A.R. El-Assy, S.H. Kandil and M.K. Kabil, *Environ. Res.* **73**, 189 (1997).
7. L.S. Forst, S. Freels and V. Persky, *J. Occup. Environ. Med.* **39**,658 (1997).
8. T.N. Wu, C.Y. Shen, J.S. Lai, C.F. Goo, K.N. Ko, H.Y. Chi, P.Y. Chang and S.H. Liou, *Arch. Environ. Health.* **55**,109 (2000).

9. Z. Nikolov, *J. Fr. Oto. Rhino. Laryngol. Audiophonol. Chir. Maxillofac.* **23**, 231 (1974).
10. K.M. Pawlas, In: Abstract book of 8th International Symposium *"Neurobehavioral Methods and Effects in Occupational and Environmental Health"* Brescia, Italy, 65 June 23-26, (2002).
11. P. Urban, E. Lukas, L. Benicky and E. Moscovicova, *Neurotoxicology.* **17**, 191 (1996).
12. L.T. Kurland, S.N. Faro and H. Siedler, *World Neurol.* **1**, 370 (1960).
13. V. Bencko and K. Symon, *Environ. Res.* **13**, 378 (1977).
14. V. Bencko, K. Symon, V. Chladek and J. Pihrt,. Environ. Res. **13**, 386 (1977).
15. J. Schwartz and D. Otto, *Arch. Environ. Health.* **46**, 300 (1991).
16. K. Osman, K. Pawlas, A. Schutz, M. Gazdzik, J.A. Sokal and M. Vahter, *Environ. Res.* **80**, 1 (1999).
17. S.A. Counter, L.H. Buchanan, G. Laurell and F. Ortega, *J. Neurol. Sci.* **152**, 85 (1997).
18. S.A. Counter, M. Vahter, G. Laurell, L.H. Buchanan, F. Ortega and S. Skerfving, *Environ. Health. Perspect.* **105**, 522 (1997).
19. L.H. Buchanan, S.A. Counter, F. Ortega and G. Laurell, *Acta Otolaryngol.* **119**, 652 (1999).
20. C.A. Whitworth, T.E. Hudson and L.P. Rybak, *Hear Res,* **129**, 61 (1999).
21. M. Anniko, *Acta Otolaryngol.* **82**, 70 (1976).
22. R. Lataye, P. Campo and G. Loquet, *Neurotoxicol. Teratol.* **21**, 267 (1999).
23. Y. Liu and L.D. Fechter, *Toxicol. Appl. Pharmacol.* **142**, 270 (1997).
24. T.C. Morata, D.E. Dunn, L.W. Kretschmer, G.K. Lemasters and R.W. Keith, *Scand. J. Work Environ. Health.* **19**, 245 (1993).
25. D.A. Otto and D.A. Fox, *Neurotoxicology.* **14**, 191 (1993).
26. K. Mizukoshi, Y. Watanabe, H. Kobayashi, Y. Nikano, C. Koide, S. Inomata and N. Saitho, *Acta. Otolaryngol.* suppl **468**, 353 (1989).
27. E.M. Jr Walker, M.A. Fazekas-May and W.R. Bowen, *Clin. Lab. Med.* **10**, 323 (1990).
28. D.C. Rice, *Toxicol. Sci,* **44**, 191 (1998).
29. D. Mergler, A.A. Boischio, F. Branches, S. Morais, C.J. Passos, E. Gaspar and M. Lucotte, In: Abstract book of 8th International Symposium *"Neurobehavioral Methods and Effects in Occupational and Environmental Health"* Brescia, Italy: p.15 June 23-26, (2002).
30. M.M. Cullen and D.A.Leopold, *Med. Clin. North Am.* **83**, 57 (1999).
31. T.J. Witek, Jr., *Am. J. Ind. Med.* **24**, 649 (1993).
32. E. Baatrup, *Comp. Biochem. Physiol. C.* **100**, 253 (1991).
33. L. Hernadi, *Neurobiology.* **1**, 11 (1993).
34. B.J. Cowart, I.M. Young, R.S. Feldman and L.D. Lowry, *Occup. Med.* **12**, 465 (1997).
35. S.S. Schiffman, *JAMA.* **278**, 1357 (1997).
36. M. Wolfensberger, I. Schnieper and A. Welge-Lussen, *Acta Otolaryngol.* **120**, 303 (2000).
37. R.L. Doty, T. Gregor and C. Monroe, *J. Occup. Med.* **28**, 457 (1986).
38. B. Sandmark, I. Broms, L. Lofgren and C.G. Ohlson, *Scand. J. Work. Environ. Health.* **15**, 60 (1989).
39. B.S. Schwartz, D.P. Ford, K.I. Bolla, J. Agnew, N. Rothman and M.L. Bleecker, *Am. J. Ind. Med.* **18**, 697 (1990).
40. C.S. Rose, P.G. Heywood and R.M. Costanzo, *J. Occup. Med.* **34**, 600 (1992).

41. G. Chiappino, G. Broich, P. Mascagni and O. Picchi, *Med. Lav.* **89**, 283 (1998).
42. B. Rydzewski, W. Sulkowski and M. Miarzynska, *Int. J. Occup. Med. Environ. Health.* **11**, 235 (1998).
43. A.R. Hirsch and G. Zavala, *Occup. Environ. Med.* **56**, 284 (1999).
44. W.J. Sulkowski, B. Rydzewski and M. Miarzynska, *Acta Otolaryngol.* **120**, 316 (2000).
45. P. Hotz, A. Tschopp, D. Soderstrom, J. Holtz, M.A. Boillat and F. Gutzwiller, *Int. Arch. Occup. Environ. Health.* **63**, 525 (1992).
46. R.L. Doty, P. Shaman, C.P. Kimmelman and M.S. Dann, *Laryngoscope.* **94**, 176 (1984).
47. W.S. Cain, J.F. Gent, R.B. Goodspeed and G. Leonard, *Laryngoscope.* **98**, 83 (1988).
48. A. Pruszewic, *Otolaryng. Pol.* **19**, 29 (1965).
49. P. Mascagni, D. Consonni, G. Bregante, A. Baj, P. Fiorillo, G. Chiappino and F. Toffoletto, In: Abstract book of 8th International Symposium "*Neurobehavioral Methods and Effects in Occupational and Environmental Health*" Brescia, Italy: p. 121 Jun 23-26, (2002).
50. R.L. Doty, A. Marcus and W.W. Lee, *Laryngoscope.* **106**, 353 (1996).
51. R.L. Doty (ed), *Handbook of Olfactation and Gustation.* New York: Marcel Dekker. 1995; 177.
52. J.E. Amoore. In C.S. Barrow (ed): *Toxicology of the nasal passages.* Washington (DC): Hemisphere Publishing, 1986; 155.
53. L. Hastings and M.L. Miller, In: *Taste and smell disorders.* A.M. Seiden, (ed). New York: Thieme Medical Publishers, 1997; 88.
54. L. Friberg, *Acta Med. Scand.* **138**, 1 (1950).
55. R.G. Adams and N. Crabtree, *Br. J. Ind. Med.* **18**, 216 (1961);.
56. C.L. Potts, *Ann. Occup. Hyg.* **8**, 55 (1965).
57. Y.Z. Liu, J.X. Huang, C.M. Luo, B.H. Xu and C.J. Zhang, *Scand. J. Work. Environ. Health.* **11** Suppl 4, 29 (1985).
58. H.J. Duncan and D.V. Smith, In: *Handbook of Olfactation and Gustation.* R.L. Doty (ed), New York: Marcel Dekker, 1995; 345.
59. L. Klimek, A. Muttray, B. Moll, J. Konietzko and W. Mann, *Laryngorhinootologie.* **78**, 620 (1999).
60. A.J. Suruda, *J. Occup. Environ. Med.* **42**, 337 (2000).
61. E.A. Emmett. *Br. J. Ind. Med.* **33**, 196 (1976).
62. L. Fornazzari, D.A. Wilkinson, B.M. Kapur and P.L. Carlen, *Acta Neurol. Scand.* **67**, 319 (1983).
63. R.I. Henkin, *JAMA.* **264**, 2803. (1990).
64. T. Yasugi, T. Kawai, K. Mizunuma, R. Kishi, I. Harabuchi, J. Yuasa, T. Eguchi, R. Sugimoto, K. Seiji and M. Ikeda, *Int. Arch. Occup. Environ. Health.* **65**, 343 (1994).
65. R.L. Doty, D.A. McKeown, W.W. Lee and P. Shaman, *Chem. Senses.* **20**, 645 (1995).
66. J. Babitch. In S.C. Bondy, K.N. Prasad (ed): *Metal Neurotoxicity.* Chicago, Ill: CRC Press, 1988; 141.
67. F.W. Sunderman, Jr., *Ann. Clin. Lab. Sci.* **31**, 3 (2001).
68. D.H. Mc Burney, In: *Handbook of Physiology. Section I: the nervous system, Vol III.* J.M. Brookhart and V.B. Mountcastle (eds). American Physiological Society, Bethesda, Maryland, 1984; 1066.

69. S.S. Schiffman In: *Smell and Taste in Health and Disease.* T.V. Getchell, R.L. Doty, L.M. Bartoshuk, J.B. Snow, Jr. (eds), New York: Raven Press, 1991; 845.
70. A.E. Mott and D.A. Leopold, *Med. Clin. North Am.* **75**, 1321 (1991).
71. J.F. Gent, M.E. Frank and A.E. Mott, In: *Taste and Smell Disorders.* A.M. Seiden, (ed). New York: Thieme Medical Publishers, 1997; 146.
72. J.C. Stevens, L.A. Cruz, J.M. Hoffman and M.Q. Patterson, *Chem. Senses.* **20**, 451 (1995).
73. H. Seeber and R. Fikentscher, *Z. Gesamte. Hyg.* **36**, 33 (1990).
74. R.I. Henkin, In *Clinical Implication of Air Pollution Research.* A.J. Finkel, W.C. Duel (eds). Acton, Mass, 1975; 193.
75. R.I. Henkin, *JAMA.* **270**, 1369 (1993).
76. C.W. Armstrong, L.W. Moore, Jr., R.L. Hackler, G.B. Miller, Jr. and R.B. Stroube, *J. Occup. Med.* **25**, 886 (1983).
77. R.C. Shoemaker, In: Abstract book of 8th International Symposium *"Neurobehavioral Methods and Effects in Occupational and Environmental Health"* Brescia, Italy: p.122 June 23-26, (2002).
78. R.A. Pevzner, L. Hernadi and J. Salanki, *Acta Biol. Hung.* **37**, 159 (1986).
79. V. Borovyagin, L. Hernadi and J. Salanki, *Acta Biol. Hung.* **40**, 237 (1989).
80. K. Iwasaki and M. Sato, *Physiol. Behav.* **32**, 803 (1984).
81. B.P. Halpern, *Environ. Health Perspect.* **44**, 101 (1982).
82. I. Darian-Smith, In: *Handbook of Physiology. Section I: the nervous system, Vol III.* J.M. Brookhart and V.B. Mountcastle (eds), American Physiological Society, Bethesda, Maryland, .1984; 739-41.
83. R. Johansson and A. Valbo, *Trends Neurosci.* **6**, 27 (1983).
84. J.M. Goldberg and U. Lindblom, *J. Neurol. Neurosurg. Psychiatry.* **42**, 793 (1979).
85. F.E. Gerr and R. Letz, *Br. J. Ind. Med.* **45**, 635 (1988).
86. O. Aaserud, J. Juntunen and E. Matikainen, *Acta Neurol. Scand.* **82**, 277 (1990).
87. F.E. Gerr, D. Hershman and R. Letz, *Int. Arch. Occup. Environ. Health.* **45**, 148 (1990).
88. R. Lundstrom, T. Stromberg and G. Lundborg, *Int. Arch. Occup. Environ. Health.* **64**, 201 (1992).
89. T. Kovala, E. Matikainen, T. Mannelin, J. Erkkila, V. Riihimaki, H. Hanninen and A. Aitio, *Occup. Environ. Med.* **54**, 487 (1997).
90. G. Lundborg, C. Sollerman, T. Stromberg, I. Pyykko and B. Rosen, *Scand. J. Work. Environ. Health.* **13**, 375 (1987).
91. G. Bartlett, J.D. Stewart, R. Tamblyn and M. Abrahamowicz, *Muscle Nerve.* **21**, 367 (1998).
92. T. Skov, K. Steenland and J. Deddens, *Am. J. Ind. Med.* **34**, 438 (1998).
93. P. Urban, Z. Jandak and B. Prochazka, *Centr. Eur. J. Public Health.* **3**, 234 (1995).
94. M.J. Hilz, F.B. Axelrod, K. Hermann, U. Haertl, M. Duetsch and B. Neundorfer, *J Neurol Sci.* **159**, 219 (1998).
95. C.M. Braun and M. Richer, J. *Stud. Alcohol.* **54**, 11 (1993).
96. R. McConnell, M. Keifer and L. Rosenstock, *Am. J. Ind. Med.* **25**, 325 (1994).
97. M. Nasterlack, M.C. Dietz, K.H. Frank, W. Hacke, H. Scherg, H. Schmittner, O. Stelzer, A. Zimber and G. Triebig, *Int. Arch. Occup. Environ. Health.* **72**, 205 (1999).

98. H.Y. Chuang, J. Schwartz, S.Y. Tsai, M.L. Lee, J.D. Wang and H. Hu, *Occup. Environ. Med.* **57**, 588 (2000).
99. M. Bovenzi, A. Fiorito and V. Patussi, *Med. Lav.* **81**, 22 (1990).
100. J. Greening and B. Lynn, *Int. Arch. Occup. Environ. Health.* **71**, 29 (1998).
101. D. Mergler, In: L.W. Chang and W. Slikker (eds). *Neurotoxicology: Approaches and Methods.* New York, Academic Press, 1995; 727-36.
102. M. Harada, J. Nakanishi, E. Yasoda, M.C. Pinheiro, T. Oikawa, G. de Assis Guimaraes, B. da Silva Cardoso, T. Kizaki and H. Ohno, *Environ. Int.* **27**, 285 (2001).
103. R.A. House, G.M. Liss and M.C. Wills, *Arch. Environ. Health.* **49**, 196 (1994).
104. R.A. House, G.M. Liss, M.C. Wills and D.L. Holness, *J. Occup. Environ. Med.* **38**, 123 (1996).
105. S.Y. Tsai and J.D. Chen, *Neurotoxicol. Teratol.* **18**, 463 (1996).
106. J.M. Langman, *Pathology.* **26**, 301 (1994).
107. R.Y. Demers, B.L. Markell and R. Wabeke, *J. Occup. Med.* **33**, 1051 (1991).
108. D.K. Broadwell, D.J. Darcey, H.K. Hudnell, D.A. Otto and W.K. Boyes, *Am. J. Ind. Med.* **27**, 677 (1995).
109. L. Stokes, A. Stark, E. Marshall and A. Narang, *Occup. Environ. Med.* **52**, 648 (1995).
110. L. London, V. Nell, M.L. Thompson and J.E. Myers, *Scand. J. Work. Environ. Health.* **24**, 18 (1998).
111. D.C. Cole, F. Carpio, J. Julian and N. Leon, *J. Toxicol. Environ. Health A.* **55**, 77 (1998).
112. D.A. Otto and K.H. Hudnell, *Environ. Health.* **62**, 159 (1993).
113. A.M. Geller and H.K. Hudnell, *Neurotoxicol. Teratol.* **19**, 455 (1997).
114. F. Gobba, *Neurotoxicology.* **21**, 857 (2000).
115. A. Iregren, M. Andersson and P. Nylén, *Neurotoxicology.* **23**, 719 (2002).
116. M. Swinker and W. Burke, *Environ. Health Perspect.* **110**, A120 (2002).
117. J.C. Quintyn, J. Massy, M. Quillard and G. Brasseur, *Acta Ophthalmol. Scand.* **77**, 23 (1999).
118. A. Cavalleri, L. Belotti, F. Gobba, G. Luzzana, P. Rosa and P. Seghizzi, *Toxicology Letters.* **77**, 351 (1995).
119. A. Cavalleri and F. Gobba, *Environ. Res.* **77**, 173 (1998).
120. P. Urban, F. Gobba, J. Nerudova, E. Lukas, Z. Cabelkova and M. Cikrt, In: Abstract book of 8$^{th}$ International Symposium *"Neurobehavioral Methods and Effects in Occupational and Environmental Health"* Brescia, Italy: p.71 June 23-26, (2002).
121. H. Langauer Lewowicka and Z. Kazibutowska, *Pol. J. Occup. Med.* **2**, 192 (1989).
122. D.G. Ellingsen, T. Morland, A. Andersen and H. Kjuus, *Br. J. Ind. Med.* **50**, 736 (1993).
123. P. Urban, E. Lukas, J. Nerudova, Z. Cabelkova and M. Cikrt, *Eur. J. Neurol.* **6**, 571 (1999).
124. H.K. Hudnell, I. Skalik, D. Otto, D. House, P. Subrt and R. Sram, *Neurotoxicology.* **17**, 615 (1996).
125. J. Lebel, D. Mergler, M. Lucotte, M. Amorim, J. Dolbec, D. Miranda, G. Arantes, I. Rheault and P. Pichet, *Neurotoxicology.* **1**, 157 (1996).
126. J. Lebel, D. Mergler, F. Branches, M. Lucotte, M. Amorim, F. Larribe and J. Dolbec, *Environ. Res.* **79**, 20 (1998).
127. L. Altmann, K. Sveinsson, U. Krämer, M. Weishoff-Houben, M. Turfeld, G. Winneke and H. Wiegand, *Neurotoxicol Teratol.* **20**, 9 (1998)

128. R. Lucchini, E. Albini, I. Cortesi, D. Placidi, E. Bergamaschi, F. Traversa and L. Alessio, *Neurotoxicology.* **21**, 805 (2000).
129. C. Abbate, R. Buceti, F. Munao, C. Giorgianni andG. Ferreri, *Int. Arch. Occup. Environ. Health.* **66**, 389 (1995)
130. K. Murata, S. Araki, K. Yokoyama, K. Nomiyama, H. Nomiyama, Y.X. Tao and S.J. Liu, *Am. J. Ind. Med.* **28**, 233 (1995).
131. S.J. Rothenberg, L. Schnaas, V.M. Salgado, E. Casanueva, A.M. Geller, H.K. Hudnell and D.A. Fox, *Invest. Ophthalmol. Vis. Sci.* **43**, 2036 (2002).
132. H. Sinczuk-Walczak, M. Jakubowski and W. Matczak, *Int. J. Occup. Med. Environ. Health.* **14**, 329 (2001).
133. F. Gobba, C. Galassi, M. Imbriani, S. Ghittori, S. Candela and A. Cavalleri, *J. Occup. Med.* **33**, 761 (1991).
134. F. Gobba and A. Cavalleri, *Neurotoxicology.* **21**, 777 (2000).
135. G. Triebig, T. Stark, A. Ihrig and M.C. Dietz, *J. Occup. Environ. Med.* **43**, 494 (2001).
136. L. Castillo, M. Baldwin, M.P.Sassine and D. Mergler, *Am. J. Ind. Med.* **39**, 351 (2001).
137. T.A. Mirzoev and M.Y. Sultanov, *Oftalmol. Zh.* **5**, 262 (1989).
138. P. Carricaburu, R. Lacroix and J. Lacroix, *Ann. Pharm. Fr.* **38**, 155 (1980).
139. M.V. Vettori, D. Corradi, T. Coccini, A. Carta, S. Cavazzini, L. Manzo and A. Mutti, *Neurotoxicology.* **21**, 607 (2000).
140. H.H. Schaumburg and P.S. Spencer, *Science.* **199**,199 (1978).
141. K.M. Crofton, D. Ding, R. Padich, M. Taylor and D. Henderson, *Hear. Res.* **144**, 196 (2000).
142. C. Till, C.A. Westall, J.F. Rovet and G. Koren, *Teratology.* **64**, 134 (2001).
143. C. Till, G. Koren, J.F. Rovet and C.A. Westall, In: Abstract book of 8th International Symposium "*Neurobehavioral Methods and Effects in Occupational and Environmental Health*" Brescia, Italy June 23-26, (2002).
144. P. Nylen, M. Hagman and A.C. Johnson, *Pharmacol. Toxicol.* **74**, 116 (1994).
145. R. Cary, S. Clarke and J. Delic, *Ann. Occup. Hyg.* **41**, 455 (1997).
146. B.W. Blakeley and S.F. Myers, *Otolaryngol. Head. Neck. Surg.* **109**, 385 (1993).
147. L.D. Fechter and L. Carlisle, *Toxicol. Appl. Pharmacol.* **105**, 133 1990)

